# Correlation between Morphological and Hemodynamic Parameters of Carotid Arteries and Cerebral Vasomotor Reactivity

**DOI:** 10.3390/brainsci14020167

**Published:** 2024-02-07

**Authors:** Stefan Stoisavljevic, Milica Stojanovic, Mirjana Zdraljevic, Vuk Aleksic, Tatjana Pekmezovic, Milija Mijajlovic

**Affiliations:** 1 Faculty of Medicine, University of Belgrade, 11000 Belgrade, Serbia; stoisavljevic.stefan90@gmail.com (S.S.); pekmezovic@sezampro.rs (T.P.); 2 Neurology Clinic, University Clinical Center of Serbia, 11000 Belgrade, Serbia; milica.stojanovic90@yahoo.com (M.S.); arsenijevicmirjana0905@gmail.com (M.Z.); 3 Neurosurgery Department, Clinical-Hospital Center Zemun, 11000 Belgrade, Serbia; aleksicvuk@hotmail.com

**Keywords:** carotid atherosclerosis, carotid stenosis, cerebral vasomotor reactivity, breath holding index, vascular risk factors

## Abstract

The function of cerebral small vessels can be assessed using cerebral vasomotor reactivity (VMR). Our aim in this retrospective cross-sectional study was to investigate a correlation between carotid artery stenosis measured through ultrasonographic morphological and hemodynamic parameters and cerebral VMR. A total of 285 patients (125 males; mean age 54) were included. The breath-holding index (BHI) was used to evaluate cerebral VMR. Ultrasonographic carotid artery parameters were collected: the presence and characteristics of carotid plaques, the degree of carotid diameter stenosis, intima–media thickness (IMT), peak systolic velocity (PSV), and end diastolic velocity (EDV). Additionally, hemodynamic parameters of the middle cerebral artery (MCA) were evaluated, including the mean flow velocity (MFV) and pulsatility index (PI). The following was collected from patients’ medical histories: age, gender, and vascular risk factors. A negative correlation between the BHI and age (r = −0.242, *p* < 0.01), BHI and the presence of carotid plaques, BHI and IMT (r = −0.203, *p* < 0.01), and BHI and the PI of MCA on both sides (r = −0.268, *p* < 0.01) was found. We found a positive correlation between the BHI in the left MCA and EDV in the left internal carotid artery (r = 0.121, *p* < 0.05). This study shows the correlation between cerebral VMR and carotid stenosis but indicates a higher influence of morphological parameters on VMR values.

## 1. Introduction

Cerebral autoregulation is the ability of cerebral blood vessels to regulate blood flow to satisfy metabolic and hemodynamic needs by changing the diameter of small blood vessels, arterioles, and precapillary sphincters. This mechanisms’ purpose is to maintain the perfusion of the brain and provide oxygen to the brain consistently. Cerebral blood flow values are usually independent of systemic blood pressure [1,2,3]. 

Vasomotor reactivity (VMR) represents a change in cerebral blood flow according to the increased partial pressure of arterial carbon dioxide (CO_2_). An increase in the partial pressure of CO_2_ (hypercapnia) causes vasodilation, which increases cerebral blood flow. Small arterioles of the brain are very sensitive to arterial CO_2_ so this mechanism can be activated by breath holding [1,2]. VMR provides significant information about cerebral hemodynamic status. Transcranial Doppler (TCD) is a non-invasive real-time method which can be used for VMR evaluation during breath holding (apnea test) by measuring breath-holding index (BHI) [1,4].

In chronically reduced cerebral blood supply, as seen in carotid artery stenosis, blood vessels are persistently dilated as a compensatory mechanism of autoregulation. In such a condition, blood vessels have less ability to dilate due to breath-holding-induced hypercapnia, which means the BHI is lower. Even though this correlation is logical, it has not been extensively studied, and the data on it are limited [1,4,5]. 

Impaired VMR has been previously correlated with conditions associated with the impaired function of cerebral small vessels such as cerebral amyloid angiopathy, as well as mild cognitive impairment and Alzheimer’s disease [6,7]. Interestingly, it was not correlated with Parkinson’s disease, suggesting that it is a good measure of cerebral small vessel function [8]. The findings of the Rotterdam study show that an impaired VMR is associated with increased mortality, specifically associated with cardiovascular mortality, independently of stroke, suggesting that a decreased VMR indicated accumulated vascular damage in these patients [9]. 

Most commonly, carotid artery stenosis represents a consequence of atherosclerosis and is directly connected with vascular risk factors including advanced age, diabetes mellitus, hypertension, hyperlipidemia, obesity, and smoking. Carotid artery stenosis is a well-known cause of ischemic stroke and is even recognized as the most common individual cause of these events. Stenosis or the occlusion of the extracranial part of internal carotid arteries causes about 10% of all ischemic strokes [10]. The risk of ischemic stroke is strongly connected to the degree of stenosis and characteristics of plaque. Furthermore, carotid stenosis has been linked to impaired cerebral autoregulation, while cerebral blood flow was restored after endarterectomy and carotid artery stenting, showing how significant this association is [5,11]. Another important prognostic ischemic stroke factor in patients with carotid artery stenosis is vasomotor reactivity, which is measured by BHI [12].

Our study aimed to correlate VMR measured by the breath holding test with morphological parameters (the presence of plaque, its characteristics, and intima–media thickness (IMT)) and the hemodynamic parameters (peak systolic velocity (PSV) and end diastolic velocity (EDV)) of the carotid arteries, in addition to the hemodynamic parameters (mean flow velocity (MFV) and pulsatility index (PI)) of the middle cerebral arteries.

## 2. Materials and Methods

In this retrospective cross-sectional study, we investigated the correlation between cerebral VMR, measured by the BHI during the breath holding test and the ultrasonographic findings of carotid and middle cerebral arteries, including the morphology and parameters.

All patients treated at Neurology Clinic, University Clinical Centre of Serbia, in Belgrade from October 2011 to December 2016 who underwent the breath holding test were considered for this study. Patients who were unable to undergo the breath holding test either due to any known cardiopulmonary diseases, contraindications to exercise, the use of medication known to influence vascular function, the inability to hold their breath for 30 s, or the decreased transparency of temporal bone from one or both sides were excluded from the study. Of the original 369 patients considered for this study, 285 patients were included. Patients’ medical histories were then analyzed from their medical records using the hospital information system—Infomedis^®^.

### 2.1. Demographics and Risk Factors

Demographic data (age and gender) as well as data on vascular risk factors, including hypertension, diabetes mellitus, atrial fibrillation, cardiomyopathy, dyslipidemia, and smoking, were collected from medical records. 

### 2.2. Carotid and Middle Cerebral Artery Parameters 

An ultrasound examination on the extracranial carotid arteries was performed with Aloka Prosound Alpha 10 ultrasound equipment (Aloka, Tokyo, Japan), with the linear transducer set to 5–13 MHz. The following morphological and hemodynamic parameters were noted:

1. The presence and characteristics of carotid arteries plaques and the degree of carotid artery stenosis per combined criteria [13,14,15].
2. The presence of unstable plaques, defined as hypoechogenic or ulcerated, or plaques with the presence of hemorrhage upon ultrasound examination [13,14,15].
3. IMT (mm) measured by Mannheim Consensus in the distal ACC segment immediately before the bifurcation or 10 mm proximally if there are plaques in the bulbs [16].
4. The PSV and EDV of internal carotid arteries (ICAs) [13,14,15].
5. The MFV and PI of both MCAs at a depth of 55 mm [14,17].

### 2.3. Breath Hold Index 

Cerebral VMR was measured by the breath holding test and analyzed from the middle cerebral artery (MCA) on both sides through the temporal bone window, at a depth of 55 mm, using 2 MHz TCD ultrasonic transducer (Rimed Ltd., Ra’anana, Israel). Before starting the test, patients were instructed on how to behave during the test. Patients were investigated in a supine position. After normal inhalation, they were asked to hold their breath for 30 s. Their arterial blood pressure and heart rate were continuously measured during the test. Ultrasonic transducers were set on the left and right side on temporal bone to continuously measure MFV and PI in both MCAs. The BHI was calculated using a standardized formula [2,18,19]. A BHI value of at least 0.69 was considered normal. Values less than 0.69 are considered pathological and show that cerebral VMR is decreased [5,18].

### 2.4. Statistical Analysis 

The Kolmogorov–Smirnov test was used to test the data for the normality of distribution. Categorical variables were reported as absolute numbers and percentages, while continuous variables were reported as a mean and standard deviation (SD) or median and interquartile range (IQR). Based on the normality of distribution as well as the type of data, appropriate statistical tests were used. Differences between groups were evaluated with the Pearson χ^2^ test or Fisher’s exact test for categorical variables and using Student’s *t* test and Mann–Whitney U test for continuous variables. Analysis of variance was used to test differences between groups. We accepted the statistical significance of differences if *p* < 0.05, or in especially emphasized situations, *p* < 0.01. The statistical analysis was performed using IBM SPSS Statistics 29 (IBM, Armonk, New York, NY, USA).

## 3. Results

Our retrospective study included 285 patients, of whom 125 (43.85%) were males. The mean age was 54.6 ± 16.2 years. Most of our patients had vascular risk factors. Dyslipidemia was found in 153 (53.7%) patients, while 147 (51.6%) patients had hypertension, and 38 (13.3%) had diabetes mellitus. Atrial fibrillation was diagnosed in six patients (2.1%) and cardiomyopathy in eight (2.8%). Almost one-third of patients were smokers (*n* = 88, 30.9%).

The mean IMT was 0.87 ± 0.26 mm, while the average degree of stenosis in the right and left ICAs due to plaque was similar. In the right ICA, the mean degree of stenosis was 14.6% (varying from unregistered plaque to a maximum of 95%), while in left ICA, the mean degree of stenosis was 13.35% (varying from unregistered plaque to a maximum of 60%). The majority of patients had no registered stenosis. In the right ICA, plaques were not detected in 171 (60%) patients, while 108 (37.89%) had stenosis of up to 50%, and only six (2.11%) had plaque stenosis over 50%. The values were similar on the left side: 174 (61.06%) patients did not have plaques, while 108 (37.89%) had stenosis up to 50%, and only 3 (1.05%) had plaques with stenosis over 50%. More unstable plaques were detected in the left (six patients, 2.1%) than in the right ICA (two patients, 0.7%). The mean values of the BHI were also similar on both sides. In the right MCA, the mean BHI was 1.37 (0.23–4.10), while a mean BHI of 1.42 (0.25–5.10) was noted in the left MCA. A one-sample Kolmogorov–Smirnov test of homogeneity has shown a normal distribution of the BHI (Kolmogorov–Smirnov Z value of the BHI of the right side = 1.761, and of the BHI of the left side = 1.577). Table 1 shows the hemodynamic parameters in the internal carotid arteries and middle cerebral arteries. 

The statistical correlation between the BHI measured in the MCA and demographic characteristics of our cohort are presented in Table 2. The Pearson Correlation test has determined a negative correlation between age and the BHI value on both sides of the MCA (right r = −0.242; left r = −0.240, *p* < 0.01 on both sides). Namely, the BHI values are decreasing with age, and VMR is deteriorating accordingly. The ANOVA test showed a statistically significant correlation between hypertension and dyslipidemia with reduced BHI values for both left and right MCA. In our study, hypertension and dyslipidemia correlated with a higher degree of stenosis (*p* < 0.01). Due to the suspicion of a confounding effect of carotid stenosis and vascular risk factors in our study, we stratified the data based on the level of carotid stenosis (no stenosis; stenosis up to 50%; and stenosis of more than 50%). After stratification, no correlation was found between hypertension and dyslipidemia separately with a reduced BHI (*p* > 0.05 for all groups). 

Table 3 shows the correlation between BHI values on one end and morphological and hemodynamic parameters of carotid arteries on the other end. The correlation between the BHI and IMT was examined by the Pearson Correlation test, and a statistically significant negative correlation between the IMT and BHI values was found. There is a connection between higher IMT values and lower BHI values (right r = −0.203; left r = −0.168, *p* < 0.01 for both). A statistical correlation between BHI and morphological characteristics other than the IMT was determined by nonparametric correlations (Spearman’s rho test). A negative correlation between the BHI and the existence of plaque in the ipsilateral ICA was found (right r = −0.258; left r = −0.191, *p* < 0.01 for both). The existence of plaques in carotid arteries is associated with lower BHI values in both the ipsilateral MCA and contralateral MCA (right r = −0.221; left r = −0.203, *p* < 0.01 for both). From the hemodynamic characteristics, there was a statistically significant positive correlation only between the BHI values of the left MCA and the EDV of the left ICA (r = 0.121, *p* < 0.05), while other correlations were not statistically significant.

The correlation between the BHI and hemodynamic MCA parameters was determined by the Pearson Correlation test (Table 4). There was a negative correlation between the BHI and PI values of the ipsilateral (right r = −0.268, left r = −0.190, *p* < 0.01) and contralateral side (right r = −0.234, left r = −0.202, *p* < 0.01). There was no statistically significant correlation between the MFV of the MCA and BHI values.

## 4. Discussion

In this study, we showed the existence of a correlation between cerebral VMR and the ultrasonographic morphological and hemodynamic parameters of carotid arteries. Our findings indicate a higher influence of morphological parameters of carotid arteries on cerebral VMR than hemodynamic ones. 

Hypertension and dyslipidemia are known risk factors for carotid stenosis and have been shown to increase its prevalence [20]. In our study we initially found a correlation between hypertension and dyslipidemia and pathological BHI values; however, after stratification based on the degree of carotid stenosis, no correlation was found between hypertension and dyslipidemia independently of the BHI across all groups (*p* > 0.05). The study of Giannopoulos et al. showed a connection between metabolic syndrome (hypertension, dyslipidemia, diabetes mellitus, and obesity), and a reduced VMR, but the impact of individual metabolic syndrome components and an impaired VMR was not investigated. Importantly, they did not find a correlation between cerebral VMR and other relevant demographic and clinical data including age, sex, age, and race, as well as the presence of cardiac disease or small vessel disease, or the use of statin therapy [21]. Recently, Shimabukuro et al. correlated endothelial dysfunction, which is necessary for normal VMR, with individual metabolic risk components. They showed an even stronger relation between endothelial dysfunction and clustered metabolic syndrome components, especially under a condition with low insulin sensitivity [22]. Other authors found no correlation between hypertension or dyslipidemia and the BHI, which coincides with our findings after stratification [14,15,19]. We found a negative correlation between age and the BHI. This is contrary to the results of previously mentioned studies [14,15,17]. It is possible that this correlation is specific for our patients because 51.6% of them had hypertension, and 53.7% had dyslipidemia; these diseases’ frequency increases with age, so they probably manifest their cumulative proatherosclerotic effect.

The correlation between diabetes and a low BHI was previously shown, but we found no such correlation. Kozera et al., in their study, showed that a low BHI is correlated with diabetes type 1 and not IMT. Even though this is a relevant finding, it is not applicable to our patient population due to some differences in the study design. Kozera et al. focused on type 1 diabetes in patients without a history of cerebrovascular events with a mean age 32. Our cohort consisted of significantly older patients who more commonly had type 2 diabetes. As diabetes takes years to present its effect on cerebral blood vessels, it is possible that our patients have not been exposed to this disorder for a long enough period of time for it to take effect and demonstrate the dysfunction of small cerebral blood vessels [23]. 

In our cohort, the mean degree of diameter stenosis in the right and left ICA was similar (14.6% vs. 13.35%) but plaques were more stable in the right ICA than the left one (0.7% vs. 2.1% of unstable plaques). This is in accordance with the current literature, as plaque distribution is not symmetrical, and plaques in the left ICA are more commonly unstable [24]. 

We found a negative correlation between the IMT and BHI. The increase in IMT is considered to be an early marker of morphological as well as functional artery changes, even when no atherosclerotic plaques have been formed [16]. There is a possibility that the decrease in VMR and initial atherosclerotic changes of blood vessels have a common etiology which would explain the correlation between the VMR reduction and increased IMT. In addition, as the IMT is a measurement of carotid atherosclerosis and a risk factor as well as a predictor of cerebrovascular disease, its negative correlation with VMR implies that there is a significant connection between large vessel atherosclerosis and cerebral small vessel function [25,26]. To our knowledge, there are no studies assessing VMR in early carotid disease, which is why the correlation between IMT and VMR is not investigated enough. Even though most of the studies focus on advanced or clinically significant carotid stenosis or even carotid occlusion and its correlation with VMR, it is only logical to assume that increased IMT as a marker of early atherosclerotic changes would correlate with small cerebral vessel function and VMR itself as they are both subjected to the same process, i.e., atherosclerosis [12,21,27]. Our study shows the possibility of this correlation, implicating that in patients with normal IMT, VMR is preserved.

Our study showed a significant correlation between carotid artery stenosis and cerebral VMR. Patients with plaques in their ICA had significantly lower BHI values, which is in accordance with previous studies [19,22,27,28]. Ju et al. showed similar findings, in which the measured VMR was lower in patients with carotid stenosis than the control group [29]. Additionally, our findings confirm the initial hypothesis that the vasodilatation of cerebral blood vessels in patients with carotid artery stenosis exist in basal conditions, and therefore, the possibility of additional dilatation under the influence of hypoxic stimuli is reduced. Bokkers et al. showed that the stenosis of one carotid artery affects VMR only ipsilaterally [28]. Additionally, Marshall et al. and Gur et al., in their separate studies, showed that patients with unilateral high-grade carotid artery stenosis have significantly lower VMR values only on the occluded side [30,31]. Our study showed that the stenosis of the carotid artery on one side is associated with a reduced cerebral VMR on both sides. A possible explanation of lower BHI values contralaterally is the existence of collateral vessels through which blood between the hemispheres redistributes, so the side with the insufficiency could be adequately perfused. In such circumstances, healthy blood vessels are constantly dilated, causing reduced VMR. 

VMR and the BHI were previously evaluated in patients with asymptomatic and symptomatic high-grade stenosis, where it was shown that it can be used to predict the risk of stroke [32]. It is important to point out that most of our patients had no carotid stenosis or had low-grade carotid stenosis, meaning less than 50%, which is not considered hemodynamically significant. The data on the effect of low-grade stenosis on VMR values are limited. In this situation, it is difficult to determine the exact correlation between carotid stenosis and VMR. Even though the clinical focus is on stenosis of 50% or greater due to the increased risk of embolic events, recent studies indicate the need for observation of low-grade carotid stenosis. Although the risk of an embolic large-to-small vessel stroke is low with low-grade carotid stenosis, Ghaznawi et al. showed a significant correlation between cortical and subcortical atrophy as well as a decline in executive functions and memory, independent of cardiovascular risk factors. Similar results were achieved by Alhusaini et al., who showed that low-grade carotid stenosis is a marker of processes that affect cerebral cortex and function. In both cases, authors concluded that low-grade carotid stenosis may be clinically important, which is in agreement with our conclusions and the correlation between increasing carotid stenosis and decreasing VMR [33,34].

In this study, there was a positive correlation between the BHI value of the left MCA and the EDV value of the left ICA, while other correlations showed no statistical significance. Since we have only shown a partial correlation, we believe that further research on a larger number of patients is required to precisely define the existence of other possible correlations; however, it should be kept in mind that only a small number of patients have demonstrated a high degree of carotid artery stenosis, which would explain these results. Published data show a correlation between a PSV value increase and a rise of ICA stenosis, to the degree of stenosis of approximately 80% when the PSV values decrease due to a significant flow reduction [17]. Therefore, it is possible for an ICA stenosis of 50–60% to show similar PSV values to those for a stenosis of 90–95%. PSV values are also influenced by the length of the stenosed segment of the blood vessel, the presence of distal stenoses (tandem lesions) and the existence of contralateral stenosis [17]. Hence, the interpretation of the PSV as a separate hemodynamic parameter has its limitations.

Our study has indicated a statistically significant negative correlation between the BHI and PI in the MCA, more specifically, patients with impaired VMR have higher PI values. Lee et al. found that MCA stenosis independently correlated with a decrease in VMR. This study used MR angiography to evaluate MCA stenosis, while we used ultrasonography to evaluate MCA stenosis [35]. Earlier studies showed that increased PI values act as a cerebral microangiopathy marker [28,36]. Thus, the results of our study confirm that the morphological changes of cerebral blood vessels correlate with functional alterations, which are represented by the BHI. These findings are in agreement with previous studies that found that there is a correlation between the VMR and PI of the MCA, indicating that the PI could provide some insight into the VMR but not enough to determine the patients’ risk of cerebral infarction [37].

### 4.1. Future Perspectives

As our study shows a significant correlation between VMR values and the morphological and hemodynamic characteristics of carotid arteries, it is logical to think that a causal correlation could exist; However, data are lacking on this front, and there are few studies assessing the risk of patients with reduced VMR developing cerebral ischemia. We believe that a prospective longitudinal follow-up study including patients’ clinical data and outcomes as well as magnetic resonance imaging findings are needed to address this issue, allowing for the identification of patients who are at high risk for cerebral ischemia. 

### 4.2. Limitations

The main limitation of this study is the cross-sectional retrospective design that did not allow for the follow-up of patients or the assessment of any future ischemic events. Additionally, it is important to point out that we set out to include all patients treated at the Neurology Clinic of the University Clinical Center of Serbia in the period between 2011 and 2016. As not all patients were able to undergo the breath holding test, the sample size was significantly reduced. 

## 5. Conclusions

The results of our study demonstrate the existence of a significant correlation among cerebral VMR and the morphologic and hemodynamic parameters of the carotid arteries. We found that morphological parameters of the carotid arteries, which reflect structural changes, have a greater impact on VMR than the hemodynamic parameters. We believe that carotid artery ultrasound, especially when there are more pronounced stenotic changes, should be supplemented with VMR testing to determine the functional changes of intracranial blood vessels. 

A detailed assessment of morphological and functional status of blood vessels enables us to identify patients who are at high risk for cerebral ischemia and recognize patients with a higher risk of recurrent stroke, leading to more adequate therapeutic strategies.

## Figures and Tables

**Table 1:** Hemodynamic parameters of carotid and middle cerebral arteries.

	Min	Max	Mean	SD
PSV ICA right	0	140	86.02	17.6
PSV ICA left	0	300	87.38	21.31
EDV ICA right	0	65	30.15	8.96
EDV ICA left	0	126	30.97	10.97
MFV MCA right	22.00	202.00	47.96	30.00
MFV MCA left	20.00	86.00	45.82	12.29
PI MCA right	0	2.00	0.82	0.24
PI MCA left	0.30	1.97	0.83	0.25

Abbreviations: PSV = peak systolic velocity (cm/sec); EDV = end diastolic velocity (cm/sec); ICA = internal carotid artery; MFV = mean flow velocity (cm/sec); PI = pulsatility index; MCA = middle cerebral artery; SD = standard deviation.

**Table 2:** Correlation between BHI measured in MCA and demographic characteristics.

		BHI Right	BHI Left
Age	r	−0.242	−0.240
	*p*	<0.01 *	<0.01 *
Gender	*p*	0.926	0.915
Hypertension (after stratification)	*p*	>0.05	>0.05
Diabetes mellitus	*p*	0.255	0.597
Dyslipidemia (after stratification)	*p*	>0.05	>0.05
Smoking	*p*	0.226	0.080
Atrial fibrillation	*p*	0.487	0.312
Cardiomyopathy	*p*	0.276	0.106

* level of statistical significance 0.01; Abbreviations: BHI = breath holding index; MCA = middle cerebral artery.

**Table 3:** Correlation between BHI values and morphological and hemodynamic characteristics of carotid arteries.

		BHI Right	BHI Left
IMT	r	−0.203	−0.168
	*p*	0.001 *	0.001 *
Carotid plaque right	r	−0.258	−0.203
	*p*	0.000 *	0.001 *
Stability of plaque right	r	−0.034	−0.044
	*p*	0.564	0.459
Carotid plaque left	r	−0.221	−0.191
	*p*	0.000 *	0.001 *
Stability of plaque left	r	−0.064	−0.117
	*p*	0.281	0.049
PSV ICA right	r	0.031	0.032
	*p*	0.599	0.586
EDV ICA right	r	0.082	0.069
	*p*	0.169	0.440
PSV ICA left	r	0.018	0.019
	*p*	0.764	0.752
EDV ICA left	r	0.072	0.121
	*p*	0.224	0.043 **

* level of statistical significance 0.01; ** level of statistical significance 0.05; Abbreviations: IMT = intima–media thickness (mm); BHI = breath holding index; ICA = internal carotid artery; PSV = peak systolic velocity; EDV = end diastolic velocity.

**Table 4:** Correlation between BHI and hemodynamic characteristics of MCA.

		BHI Right	BHI Left
MFV MCA right	r	−0.017	0.008
	*p*	0.772	0.900
PI MCA right	r	−0.268	−0.202
	*p*	0.000 *	0.001 *
MFV MCA left	r	0.027	0.093
	*p*	0.652	0.121
PI MCA left	r	−0.234	−0.190
	*p*	0.000 *	0.001 *

* level of statistical significance 0.01; Abbreviations: BHI = breath holding index; MCA = middle cerebral artery; MFV = mean flow velocity; PI = pulsatility index;.

## Data Availability

The data that support the findings of this study are available upon request from the corresponding author. The data are not publicly available due to concerns regarding the privacy of study participants.
